# Supplementary material for: Conditional KCa3.1-transgene induction in murine skin produces pruritic eczematous dermatitis with severe epidermal hyperplasia and hyperkeratosis
Source: PLoS One. 2020 Mar 9;15(3):e0222619. doi: 10.1371/journal.pone.0222619 (PMC7062274; doi:10.1371/journal.pone.0222619)
Supplement: S1 Fig — (PPTX) [file pone.0222619.s003.pptx]

## Slide 1
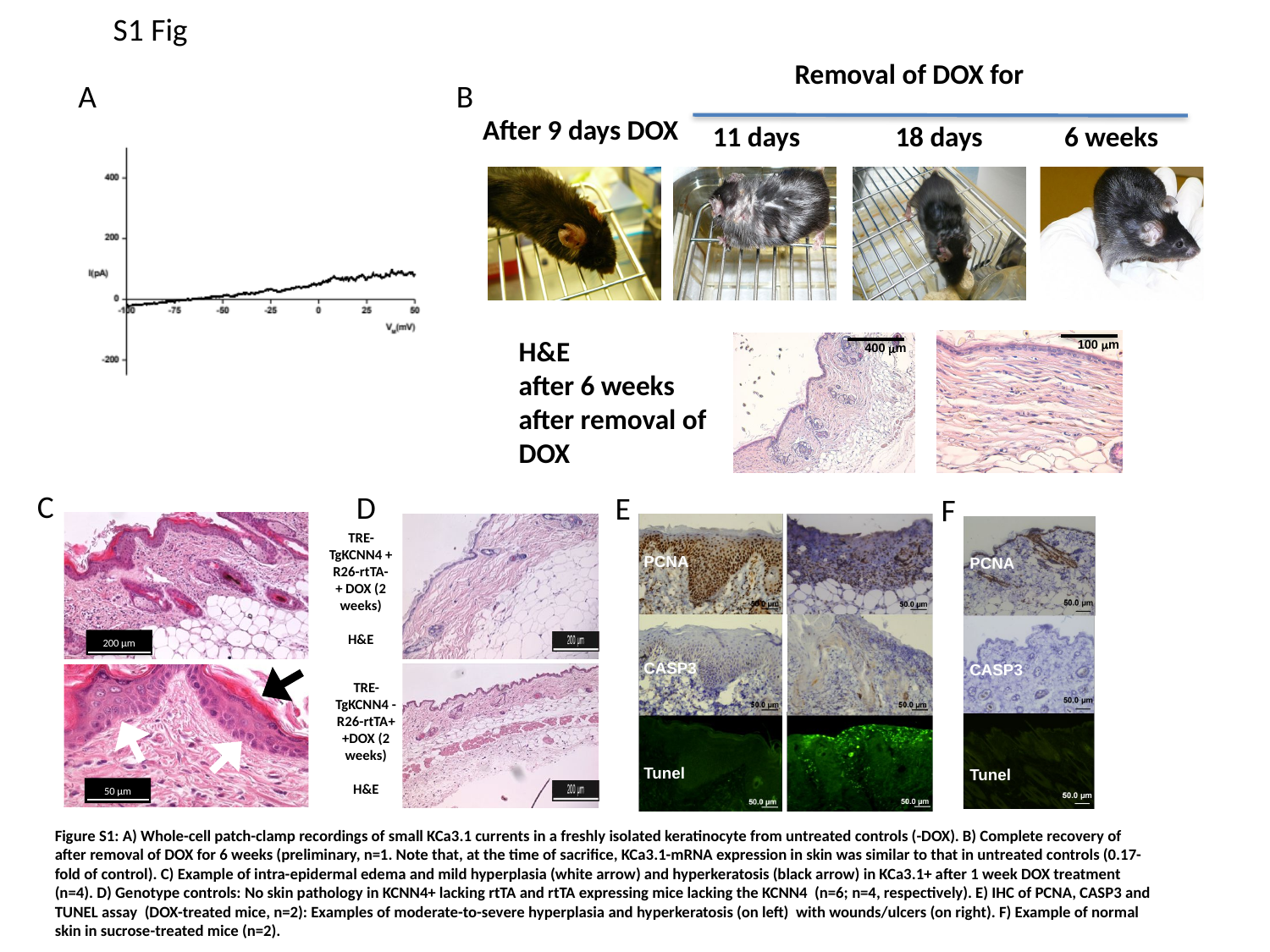

S1 Fig
Removal of DOX for
After 9 days DOX
11 days
18 days
6 weeks
H&E
after 6 weeks after removal of DOX
100 mm
400 mm
B
A
C
D
E
F
TRE-TgKCNN4 +
R26-rtTA-
+ DOX (2 weeks)
H&E
PCNA
PCNA
200 μm
CASP3
CASP3
TRE-TgKCNN4 -
R26-rtTA+
+DOX (2 weeks)
H&E
Tunel
Tunel
50 μm
Figure S1: A) Whole-cell patch-clamp recordings of small KCa3.1 currents in a freshly isolated keratinocyte from untreated controls (-DOX). B) Complete recovery of after removal of DOX for 6 weeks (preliminary, n=1. Note that, at the time of sacrifice, KCa3.1-mRNA expression in skin was similar to that in untreated controls (0.17-fold of control). C) Example of intra-epidermal edema and mild hyperplasia (white arrow) and hyperkeratosis (black arrow) in KCa3.1+ after 1 week DOX treatment (n=4). D) Genotype controls: No skin pathology in KCNN4+ lacking rtTA and rtTA expressing mice lacking the KCNN4 (n=6; n=4, respectively). E) IHC of PCNA, CASP3 and TUNEL assay (DOX-treated mice, n=2): Examples of moderate-to-severe hyperplasia and hyperkeratosis (on left) with wounds/ulcers (on right). F) Example of normal skin in sucrose-treated mice (n=2).
